# Spatial/Frontal QRS-T Angle Predicts All-Cause Mortality and Cardiac Mortality: A Meta-Analysis

**DOI:** 10.1371/journal.pone.0136174

**Published:** 2015-08-18

**Authors:** Xinlin Zhang, Qingqing Zhu, Li Zhu, He Jiang, Jun Xie, Wei Huang, Biao Xu

**Affiliations:** 1 Department of Cardiology, Affiliated Drum Tower Hospital, Nanjing University School of Medicine, Nanjing, China; 2 Department of Respiratory Medicine, Jinling Hospital, Nanjing University School of Medicine, Nanjing, China; 3 Department of Radiology, Affiliated Drum Tower Hospital, Nanjing University School of Medicine, Nanjing, China; University of Toronto, CANADA

## Abstract

**Background:**

A number of studies have assessed the predictive effect of QRS-T angles in various populations since the last decade. The objective of this meta-analysis was to evaluate the prognostic value of spatial/frontal QRS-T angle on all-cause death and cardiac death.

**Methods:**

PubMed, EMBASE, and the Cochrane Central Register of Controlled Trials were searched from their inception until June 5, 2014. Studies reporting the predictive effect of spatial/frontal QRS-T angle on all-cause/cardiac death in all populations were included. Relative risk (RR) was used as a measure of effect.

**Results:**

Twenty-two studies enrolling 164,171 individuals were included. In the combined analysis in all populations, a wide spatial QRS-T angle was associated with an increase in all-cause death (maximum-adjusted RR: 1.40; 95% confidence interval [CI]: 1.32 to 1.48) and cardiac death (maximum-adjusted RR: 1.71; 95% CI: 1.54 to 1.90), a wide frontal QRS-T angle also predicted a higher rate of all-cause death (maximum-adjusted RR: 1.71; 95% CI: 1.54 to 1.90). Largely similar results were found using different methods of categorizing for QRS-T angles, and similar in subgroup populations such as general population, populations with suspected coronary heart disease or heart failure. Other stratified analyses and meta-analyses using unadjusted data also generated consistent findings.

**Conclusions:**

Spatial QRS-T angle held promising prognostic value on all-cause death and cardiac death. Frontal QRS-T angle was also a promising predictor of all-cause death. Given the good predictive value of QRS-T angle, a combined stratification strategy in which QRS-T angle is of vital importance might be expected.

## Introduction

As one of the most commonly used diagnostic technique, ECG is available in almost all hospitals and outpatient clinics. Numerous parameters could be easily obtained from the routine 12-lead ECG. Some of these parameters, such as QT interval and ST-segment depression, carry prognostic information for cardiovascular morbidity and mortality [1,2]. Recent attention has been paid to spatial and frontal QRS-T angles—two different forms of QRS-T angle. Spatial QRS-T angle is defined as the angle between QRS- and T-wave vectors in three-dimensional space, and frontal QRS-T angle is the projection of spatial QRS-T angle onto the frontal plane [3]. QRS-T angles reflect the deviations between ventricular depolarization and repolarization, and are postulated to have promising predictive value [4,5]. Since the last decade, the prognostic effects of spatial and frontal QRS-T angles have been extensively studied both in general population and particular subpopulations [3,5–25]. A large portion of these studies showed that a wide QRS-T angle predicted a poor prognosis [9,21], but inconsistent findings existed at the same time [7,8,11]. Therefore, this meta-analysis aims to provide a clearer understanding of the impact of QRS-T angles on the risk of all-cause death and cardiac death.

## Methods

### Literature search

We sought to identify all the published studies evaluating the prognostic value of QRS-T angles on risk of all-cause death and cardiac death in all populations. From their inception until June 5, 2014, PubMed, EMBASE, and the Cochrane Central Register of Controlled Trials (CENTRAL) were systemically searched using the following search terms and key words: "QRS-T angle" OR "QRS/T angle" OR "QRS|T angle" OR "QRST angle" OR "QRSTA". Reference lists of the identified reports and relevant reviews were manually checked for potential studies not otherwise found. No restrictions on language, type of publication were imposed.

### Study selection

After reviewing all titles and abstracts, we identified reports which were potentially relevant. Thereafter, these potentially eligible articles were further reviewed in full-text to check whether they met all of the following inclusion criteria: 1) divided spatial or frontal QRS-T angles into categorical groups and evaluated their prognostic value in hard endpoints, *i*.*e*. all-cause death or cardiac death; 2) the minimum duration of follow-up was 12 months; 3) the number of participants were at least 100; 4) reported relative risks (RRs) with 95% corresponding confidence intervals (CIs) or provided raw data necessary to calculate them. Studies fulfilled all these criteria above, regardless of the population types (both general population and other particular subpopulations, such as patients with suspected coronary heart disease [CHD]), were included in the meta-analysis.

### Data collection and quality assessment

The primary endpoint was all-cause death and the secondary endpoint was cardiac death. All endpoints were defined by the investigators of each study. Study eligibility was assessed and data were extracted independently by three reviewers (XZ, QZ and LZ). Disagreements were resolved by consensus. By using a predesigned data abstraction form, the following information was recorded: the study name/first author, type of QRS-T angle, study population (general population or other particular subpopulations), year of publication, study period, number of participants, age, gender, duration of follow-up, mean QRS-T angle, and categories of QRS-T angle. Confounding factors for which were adjusted in each study were also recorded (S1 Table). Because the set of adjustments vary within studies, which were allowed in this meta-analysis, we extracted the maximum-adjusted RRs and their corresponding 95% CIs for each categorical comparison. Meanwhile, unadjusted RRs (and 95% CIs) or raw data used to calculate RRs were extracted. The quality of the included studies was assessed independently by two investigators (LZ and HJ) using the Newcastle-Ottawa Scale criteria [26].

### Statistical analysis

**Fig 1:**
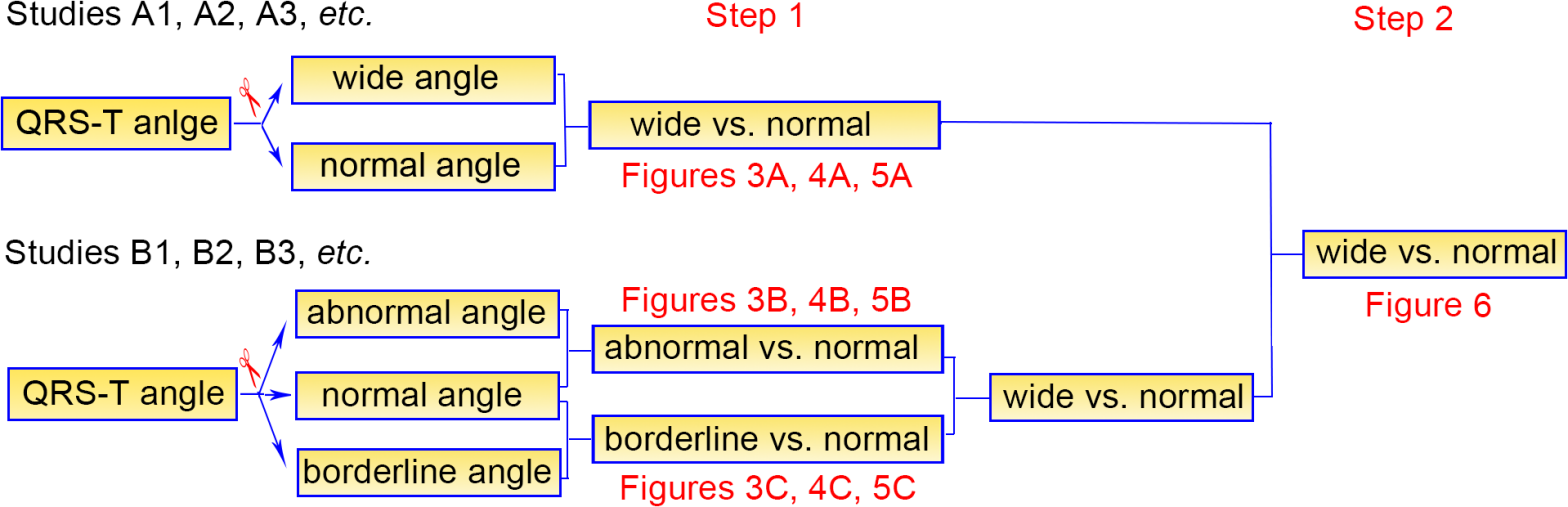
An overview of the 2-step analysis strategy in our study.

As the types of clinical presentation varied (the normal ranges of QRS-T angles varied accordingly), the cut-off points of both spatial and frontal QRS-T angles were different among these studies. As a rule, we directly employed the cut-offs defined by investigators in each individual study. A two-step analysis strategy was used in our study (Fig 1). First, in studies (A1, A2, A3, *etc*. in Fig 1) which one cutoff was set and thus QRS-T angles were divided into “wide angle” and “normal angle”, a meta-analysis of wide angles versus normal ones was conducted (step 1 in Fig 1); in studies (B1, B2, B3, *etc*. in Fig 1) which two cutoffs were set and QRS-T angles were segmented into three groups, *i*.*e*. normal, borderline and abnormal angles, we compared abnormal and borderline angles with normal angles respectively (step 1 in Fig 1). Second, data from abnormal and borderline angles were combined into wide angles in studies B1, B2, B3, *etc*., and then pooled together with data from studies A1, A2, A3, *etc*. (step 2 in Fig 1).

For all analyses, RR and its corresponding 95% CI were computed as a measure of effect. Fixed-effects models (Mantel-Haenszel method) were used to pool RR in each study unless otherwise stated [27]. The *I* ^*2*^ statistic was calculated to assess the consistency across studies, with 25% indicating low, 50% moderate, and 75% high degrees of heterogeneity [28]. Meanwhile, the χ2-based Q test was applied, a *P*>0.1 suggests significant heterogeneity [29]. In analyses with significant heterogeneity, the random-effects models (DerSimonian and Laird method) were used [30]. Publication bias was qualitatively addressed by visual inspection of the funnel plot asymmetry, as well as quantitatively assessed by Begg’s test [31]. Sensitivity analyses were carried out for all endpoints, by omitting one study at one time, to evaluate the consistency of our findings. In addition, subgroup analyses were performed by making stratifications of following factors: type of clinical presentation (general population, patients with suspected CHD, or patients with heart failure), number of participants (4000 or 2000 as cut-offs for spatial and frontal QRS-T angles respectively), and duration of follow-up (less than 5 years or more than 5 years). Meta-regression analyses were performed to estimate the interaction between prognosis (all-cause death and cardiac death) and these subgroup factors. All statistical analyses were conducted with the STATA version 11.0 (STATA Corporation, College Station, TX, USA) software. The study was performed and reported in accordance with the Preferred Reporting Items for Systematic reviews and Meta-Analyses (PRISMA) statement (S1 Checklist) [32].

## Results

### Eligible studies

**Fig 2:**
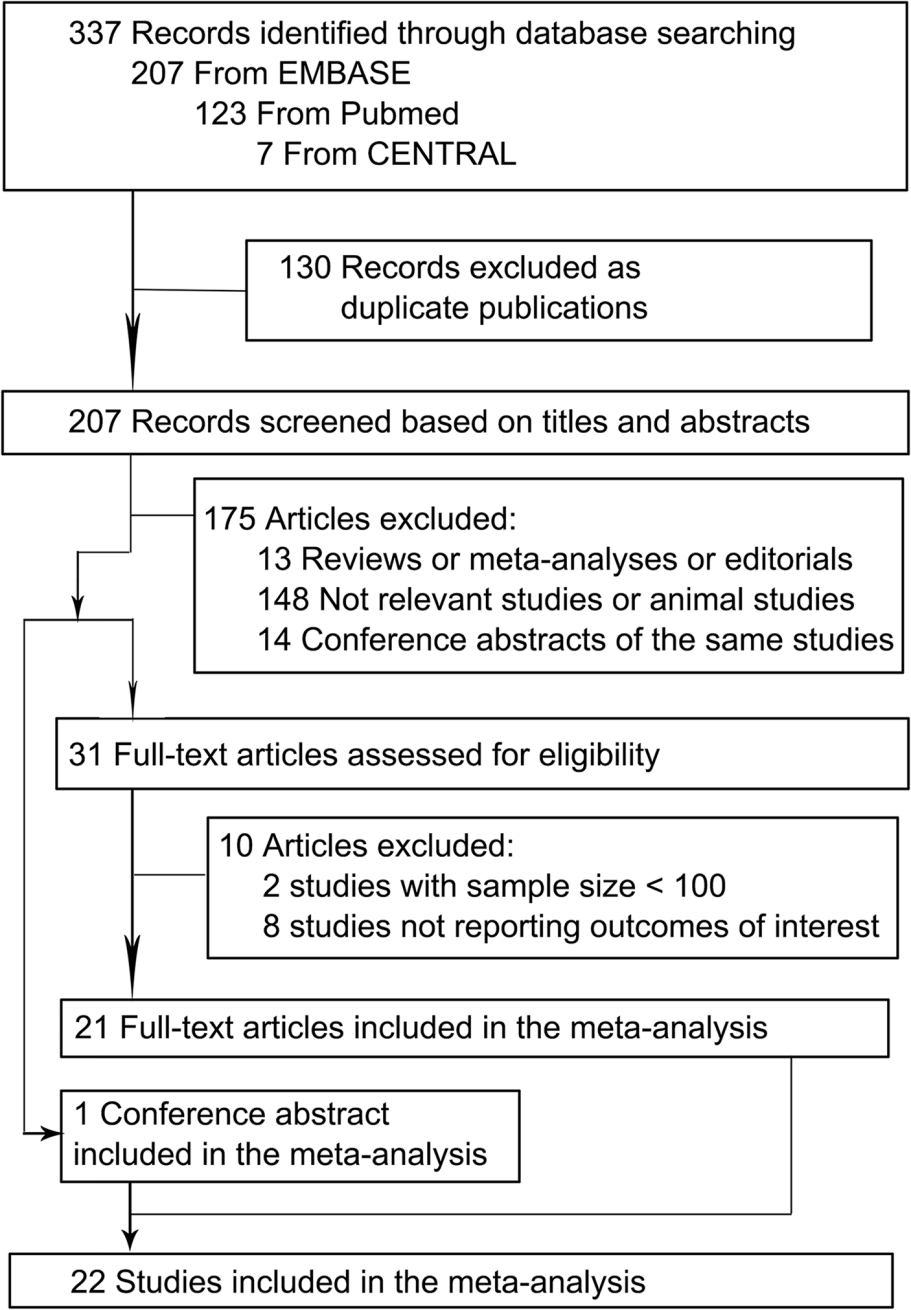
Selection of studies included in the meta-analysis.

Fig 2 shows the flow diagram of this meta-analysis. Of the 337 potentially relevant reports initially retrieved from PubMed, EMBASE and CENTRAL, 22 studies—21 full-text articles [3,5–15,17–25] and 1 conference abstract [16]—satisfied our inclusion criteria and were included in the meta-analysis. Among the 22 studies, 11 studies only reported data on all-cause death [3,7,8,10,11,13,16,17,20,23,24], 3 studies only reported data on cardiac death [12,15,21] and 8 studies reported data on both endpoints [5,6,9,14,18,19,22,25]. Ten studies were conducted in general population without a particular disease [5,9,10,12,13,19–23], while the other 12 studies were carried out in particular subpopulations, such as patients with heart failure, acute coronary syndrome, or chronic dialysis [3,6–8,11,14–18,24,25].

Table 1 summarizes the characteristics of the studies included in the meta-analysis. The period of the baseline data collection ranged from 1966 to 2011, and the duration of follow-up was between 1 and 30 years. The mean age ranged from 44 to 72 years, and the percentage of men from 0 to 100. Overall, data were available from 164171 individuals in 22 studies. In the analyses of adjusted studies, we employed data after adjustment for maximum confounders, which were listed in S1 Table. The quality of the studies was acceptable in all studies. The detailed scores of each study assessed by the Newcastle-Ottawa Scale were presented in S1 Table.

**Table 1:** Characteristics of the studies included in the meta-analysis.

Author/Study/Ref	QRS-T angle type	Population of study	Publishing Year	Period of data collection	Number of subjects	Men (%)	Age (years)	Follow-up (years)	Mean QRS-T angle (°)	Categories of QRS-T angle
**Kardys *et al*. [22]**	Spatial	Population-based	2003	1990–1993	6134	40.4	69.2±8.7	6.7	NA	≤105°, 105–135°, ≥135°
**de Torbal *et al*. [11]**	Spatial	Patients with acute ischemic chest pain	2004	1992–1994	2261	55	NA	6.3	NA	<105°, 105–135°, >135°
**Yamazaki *et al*. [21]**	Spatial	A clinical population	2005	1987–2000	46573	90	56.8±14.7	6	43.7	≤50°, 50–100°, ≥100°
**Rautaharju *et al*. (CHS) [9]**	Spatial	Over 65 years old	2006	1989–1994	4912	39.5	72.6±5.5	9.1	74±33.4	<126°, ≥126° in men; <107°, ≥107° in women
**Rautaharju *et al*. (WHI) [12]**	Spatial	Postmenopausal women	2006	1992–2007	38283	0	62.1±6.8	6.2	NA	≤56°, 57–96°; ≥97°
**Zhang *et al*. (ARIC) [10]**	Frontal	Population-based	2007	1987–1989	13873	42.3	54.4±5.7	14	23.9±24.0	≤32°, 32–73°, ≥73° in men; ≤31°, 31–67°, ≥67° in women
	Spatial								67.2±28.0	≤93°, 93–123°, ≥123° in men; ≤77°, 77–110°, ≥110°
**Pavri *et al*. (DEFINITE) [17]**	Frontal	NICM patients	2008	1998–2002	455	71.2	58.2±12.9	2.5±1.2	NA	<90°, >90°
**Borleffs *et al*. [5]**	Frontal	IHD patients with ICD therapy	2009	1996-	412	88	63±11	2±1.5	NA	<90°, >90°
	Spatial									<100°, >100°
**Lipton *et al*. [6]**	Spatial	Known or suspected CAD who underwent DSE	2009	1990–2003	2347	66	61±3	7±3.4	NA	<105°, 105–135°, >135°
**Rubulis *et al*. [15]**	Spatial	SAP patients	2010	1995–1997	187	74	58±10	8±1	NA	<101°, ≥101°
**Kentt *et al*. (FINCAVAS) [18]**	Spatial	Patients undergoing a clinically indicated bicycle stress-test	2011	2001–2007	1297	67	56±13	3.8±1	NA	<72°, ≥72°
**Aro *et al*. [4]**	Frontal	Middle-aged	2012	1966–1972	10713	52.2	43.9±8.4	30±11	20°	<90°, ≥100°
**Lown *et al*. [7]**	Frontal	ACS patients	2012	2003	1843	61.9	70.1±13.1	2	NA	≤37°, 38–104°, ≥105°
**Whang *et al*. (NHANES III) [19]**	Frontal	Over 40 years old	2012	1988–1994	7052	46.3	NA	14	NA	≤39°, 39–80°, ≥81°
	Spatial									≤90°, 90–120°, ≥121°
**de Bie *et al*. [14]**	Spatial	Chronic dialysis patients	2013	2002–2009	277	62.1	56.3±17.0	2.1±1.7	103.5±41.2	<130°, ≥130° in men; <116°, ≥116° in women
**Gotsman *et al*. [24]**	Frontal	HF patients	2013	2008-	5038	51	NA	1.6	NA	<65°, 65–124°, >124°
**Vend *et al*. (MADIT II) [16]**	Frontal	ICM patients	2013	NA	1232	NA	NA	4	NA	<90°, >90°
**Strauss *et al*. [20]**	Spatial	In and outpatients	2013	2009–2010	18488	51.8	NA	1	NA	<105°, ≥105°
**Laukkanen *et al*. [23]**	Spatial	Population-based	2014	1984–1989	1951	100	NA	20	NA	<67°, ≥67°
**Raposeiras-Roubín *et al*. [25]**	Frontal	AMI patients with depressed LVEF	2014	2004–2010	467	75.4	70±12.5	3.9	95.9±57.3	<90°, >90°
**Selvaraj *et al*. [8]**	Frontal	HFpEF patients	2014	2008–2011	376	35	64±13	1	61±51	≤26°, 27–75°, ≥76°

*Abbreviations*: ACS: acute coronary syndrome; AMI: acute myocardial infarction; ARIC: the Atherosclerosis in Communities Study; CAD: coronary artery disease; CHS: the Cardiovascular Health Study; DEFINITE: the Defibrillators in Nonischemic Cardiomyopathy Treatment Evaluation; DSE: dobutamine stress echocardiography; FINCAVAS: The Finnish Cardiovascular Study; HF: heart failure; HFpEF: heart failure with preserved ejection fraction; ICD: implantable cardioverter-defibrillator; ICM: ischemic cardiomyopathy; IHD: ischemic heart disease; LVEF: left ventricular ejection fraction; MADIT II: the Multicenter Automatic Defibrillator Implantation Trial II; NHANES III: the Third National Health and Nutrition Examination Survey; NICM: nonischemic cardiomyopathy; SAP: stable angina pectoris; WHI: The Women’s Health Initiative.

### Spatial QRS-T angle and all-cause death

**Fig 3:**
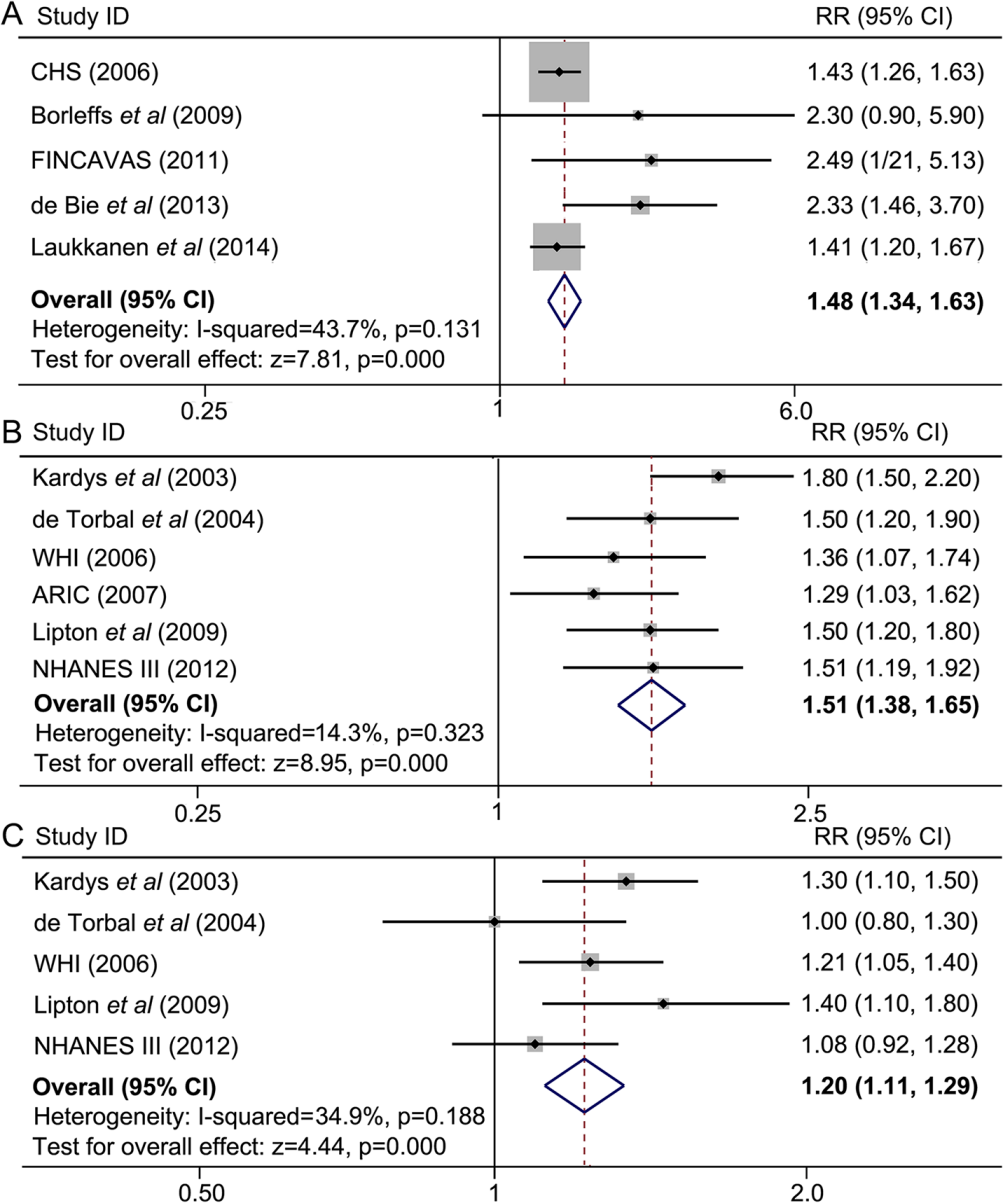
A wide spatial QRS-T angle is associated with a higher incidence of all-cause death. Meta-analyses were from separated comparisons by two methods of categorizing. (A) Spatial QRS-T angles were divided into “wide angle” and “normal angle”, a meta-analysis of wide angles versus normal ones was conducted. (B) and (C) spatial QRS-T angles were segmented into three groups, *i*.*e*. normal, borderline and abnormal angles. (B) Results from comparison between abnormal and normal, (C) results from comparison between borderline and normal. ARIC: the Atherosclerosis in Communities Study; CHS: the Cardiovascular Health Study; CI: confidence interval; FINCAVAS: The Finnish Cardiovascular Study; NHANES III: the Third National Health and Nutrition Examination Survey; RR: relative risk; WHI: The Women’s Health Initiative.

A total of 11 studies contributed to the analysis of the association between spatial QRS-T angle and all-cause death. Meta-analysis of studies A1, A2, A3, *etc*.(indicated in Fig 1) showed that a wide spatial QRS-T angle predicted a higher incidence of all-cause death (maximum-adjusted RR = 1.48, 95% CI = 1.34–1.63). No significant but a modest degree of heterogeneity was observed (*I* ^*2*^ = 43.7%, *P* = 0.131) (Fig 3A). Pooled analyses in studies B1, B2, B3, *etc*. (indicated in Fig 1) demonstrated that an abnormal spatial QRS-T angle was associated with a significantly higher mortality compared with a normal angle (maximum-adjusted RR = 1.51, 95% CI = 1.38–1.65) (Fig 3B). A weaker but still significant association was also found in borderline spatial QRS-T angles (maximum-adjusted RR = 1.20, 95% CI = 1.11–1.29) (Fig 3C). There was no evidence of significant heterogeneity.

### Spatial QRS-T angle and cardiac death

**Fig 4:**
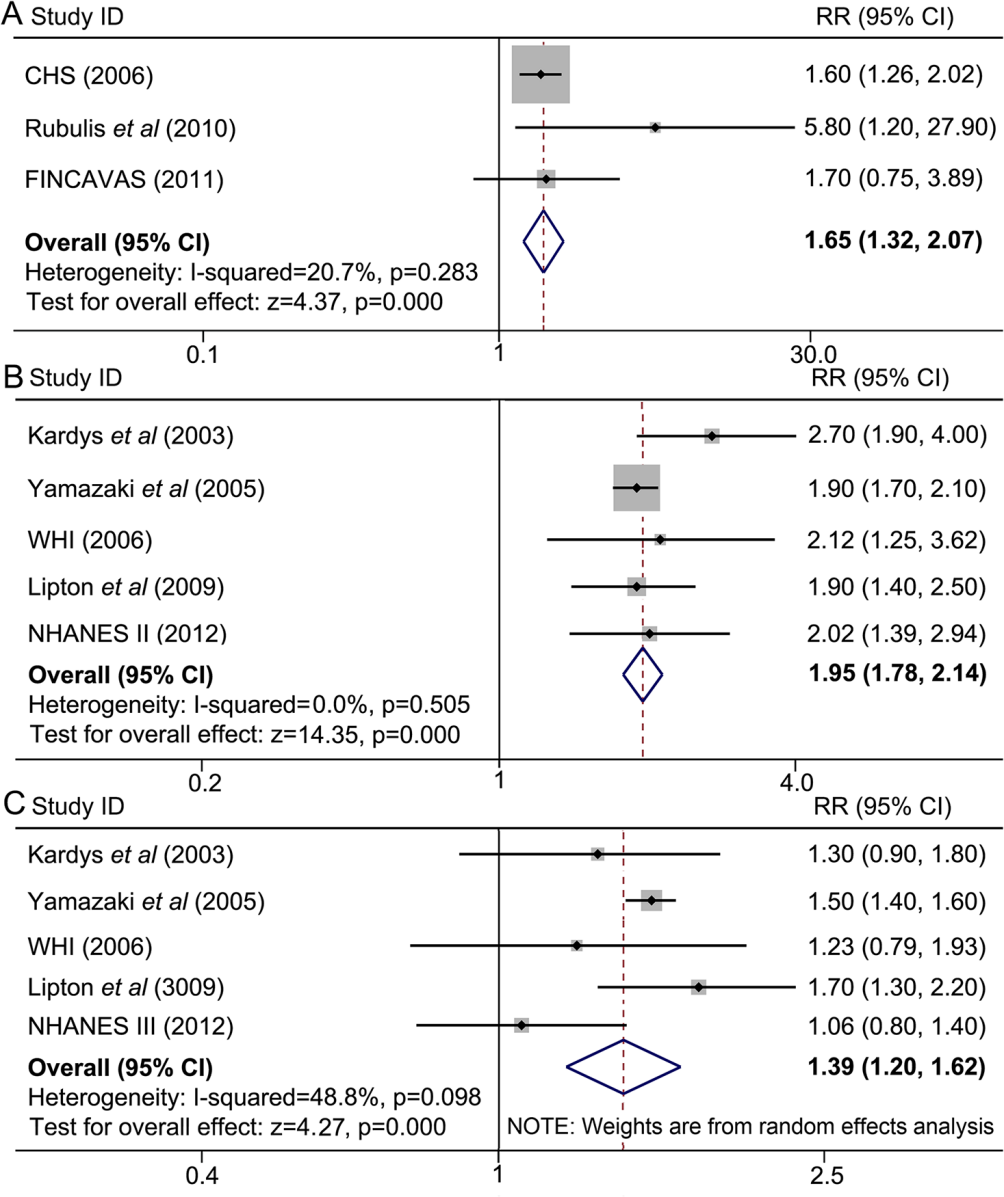
A wide spatial QRS-T angle is associated with a higher incidence of cardiac death. Meta-analyses were from separated comparisons by two methods of categorizing. (A) Spatial QRS-T angles were divided into “wide angle” and “normal angle”, a meta-analysis of wide angles versus normal ones was conducted. (B) and (C) spatial QRS-T angles were segmented into three groups, *i*.*e*. normal, borderline and abnormal angles. (B) Results from comparison between abnormal and normal, (C) results from comparison between borderline and normal. CHS: the Cardiovascular Health Study; CI: confidence interval; FINCAVAS: The Finnish Cardiovascular Study; NHANES III: the Third National Health and Nutrition Examination Survey; RR: relative risk; WHI: The Women’s Health Initiative.

A total of 8 studies contributed to the analysis of the association between spatial QRS-T angle and cardiac death. Meta-analysis of studies A1, A2, A3, *etc*. (indicated in Fig 1) showed that a wide spatial QRS-T angle predicted a higher rate of cardiac mortality (maximum-adjusted RR = 1.65, 95% CI = 1.35–2.07). No evidence of significant heterogeneity was detected (*I* ^*2*^ = 20.7%, *P* = 0.283) (Fig 4A). Pooled analyses in studies B1, B2, B3, *etc*. (indicated in Fig 1) demonstrated that an abnormal spatial QRS-T angle almost doubled the rate of cardiac mortality compared with a normal angle (maximum-adjusted RR = 1.95, 95% CI = 1.78–2.14), without evidence of heterogeneity across these studies (*I* ^*2*^ = 0%, *P* = 0.505) (Fig 4B). Similarly, a borderline spatial QRS-T angle was also associated with a higher cardiac mortality (maximum-adjusted RR = 1.39, 95% CI = 1.20–1.62), with a modest heterogeneity across studies (*I* ^*2*^ = 48.8%, *P* = 0.098) (Fig 4C).

### Frontal QRS-T angle and all-cause death

**Fig 5:**
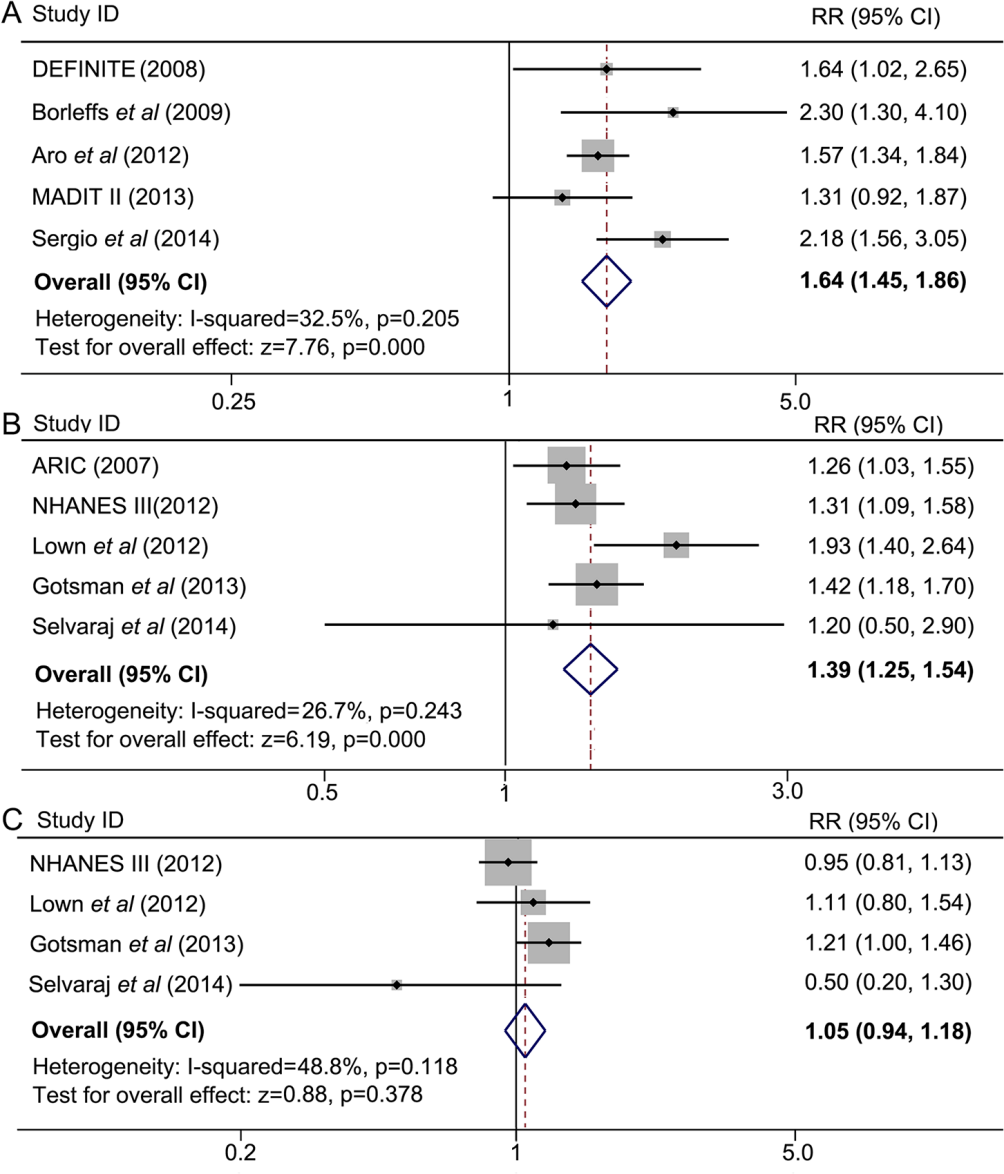
A wide frontal QRS-T angle is associated with a higher incidence of all-cause death. Meta-analyses were from separated comparisons by two methods of categorizing. (A) Frontal QRS-T angles were divided into “wide angle” and “normal angle”, a meta-analysis of wide angles versus normal ones was conducted. (B) and (C) frontal QRS-T angles were segmented into three groups, *i*.*e*. normal, borderline and abnormal angles. (B) Results from comparison between abnormal and normal, (C) results from comparison between borderline and normal. ARIC: the Atherosclerosis in Communities Study; CI: confidence interval; DEFINITE: the Defibrillators in Nonischemic Cardiomyopathy Treatment Evaluation; MADIT II: the Multicenter Automatic Defibrillator Implantation Trial II; NHANES III: the Third National Health and Nutrition Examination Survey; RR: relative risk.

A total of 10 studies contributed to the analyses of the association between frontal QRS-T angle and all-cause death. Meta-analysis in studies A1, A2, A3, *etc*. (indicated in Fig 1) showed that a wide frontal QRS-T angle was associated with a significantly higher incidence of all-cause death, the pooled maximum-adjusted RR was 1.64 and the corresponding 95% CI was 1.45 to 1.86. No evidence of significant heterogeneity was detected (*I* ^*2*^ = 32.5%, *P* = 0.205) (Fig 5A). Similar to spatial QRS-T angle, the pooled maximum-adjusted RR for all-cause death was significantly higher in participants with abnormal frontal QRS-T angles than those with normal ones (maximum-adjusted RR = 1.39, 95% CI = 1.25–1.54) (Fig 5B). However, no significant difference was observed between the borderline group and the normal group (maximum-adjusted RR = 1.05, 95% CI = 0.94–1.18) (Fig 5C). No significant heterogeneity was found across these studies.

### Combined analyses

**Fig 6:**
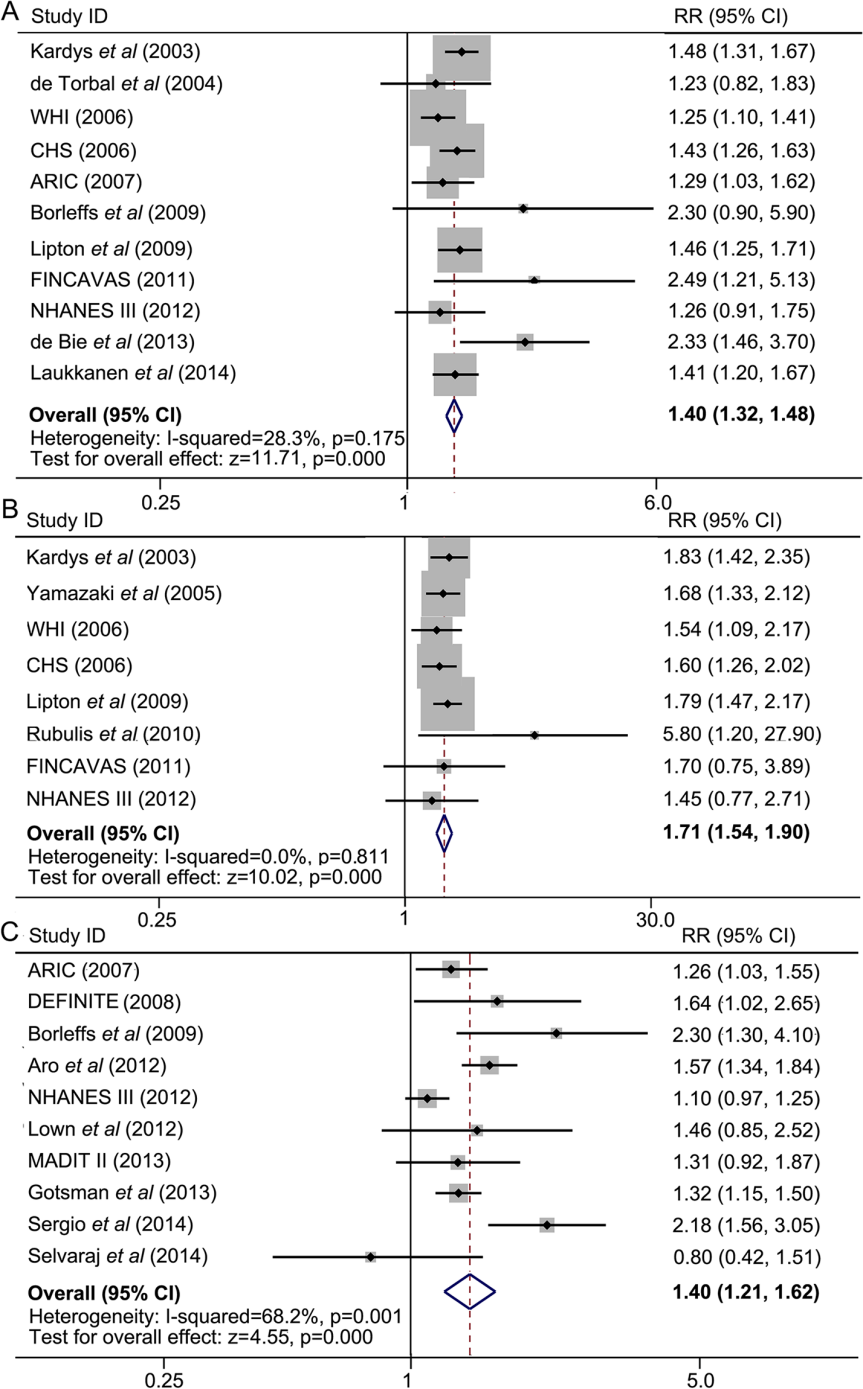
Combined analyses show that a wide spatial QRS-T angle predicts a higher incidence of all-cause death (A) and cardiac death (B), a wide frontal QRS-T angle is associated with a higher rate of all-cause death (C). ARIC: the Atherosclerosis in Communities Study; CHS: the Cardiovascular Health Study; CI: confidence interval; DEFINITE: the Defibrillators in Nonischemic Cardiomyopathy Treatment Evaluation; FINCAVAS: The Finnish Cardiovascular Study; MADIT II: the Multicenter Automatic Defibrillator Implantation Trial II; NHANES III: the Third National Health and Nutrition Examination Survey; RR: relative risk; WHI: The Women’s Health Initiative.

In step-2 analysis, we pooled together data from all studies as comparisons of wide QRS-T angles with normal angles (Fig 1). Maximum-adjusted results from 11 studies on spatial QRS-T angle and all-cause death were pooled together, and a significant positive correlation was found between a wide spatial QRS-T angle and a higher incidence of mortality (maximum-adjusted RR = 1.40, 95% CI = 1.32–1.48). No significant heterogeneity was found (*I* ^*2*^ = 28.3%, *P* = 0.175) (Fig 6A). Similarly, we pooled together maximum-adjusted data from 8 studies which reported spatial QRS-T angle and cardiac death. We found that wide spatial QRS-T angles significantly increased the rate of cardiac death, with a maximum-adjusted RR of 1.71 and corresponding 95% CI of 1.54 to 1.90. There was no evidence of heterogeneity across studies (*I* ^*2*^ = 0%, *P* = 0.811) (Fig 6B).

After pooling together all the 10 studies which reported frontal QRS-T angle and all-cause death, we found a significantly increased rate of mortality in people with wide frontal QRS-T angles (maximum-adjusted RR = 1.40, 95% CI = 1.21–1.62). However, a significant heterogeneity was also found across these studies (*I* ^*2*^ = 68.2%, *P* = 0.001) (Fig 6C). To explore the origin of heterogeneity, we carried out sensitivity analysis and stratified subgroup analyses, which would be presented in the subsequent section.

### Publication bias

With respect to all endpoints, no evidence of publication bias was found by using Begg’s test and visually inspecting the funnel plot asymmetry. As the number of studies included in part of the comparisons was limited, the qualitative evaluation by visual inspection in these analyses did not provide convincing results and the quantitative assessment by Begg’s test was used.

### Stratified and sensitivity analyses

The overall association for spatial/frontal QRS-T angles and incidence of all-cause/cardiac death remained largely consistent when these studies were stratified by several characteristics of the studies, such as the type of clinical presentation, the number of participants, and the duration of follow-up (Table 2). For instance, when comparing a wide spatial QRS-T angle with a normal one, the combined RR of all-cause death was 1.37 (95% CI = 1.29–1.46) in general population and 1.47 (95% CI = 1.28–1.65) in patients with suspected CHD. Similarly for frontal QRS-T angle, the RR was 1.29 (95%CI = 1.03–1.62) in general population, 1.74 (95%CI = 1.41–2.14) in patients with suspected CHD, and 1.51 (95%CI = 1.18–1.94) in patients with heart failure. No significant interaction was found in most subgroup analyses except that a quantitative but not qualitative interaction was observed in analysis stratified by duration of follow-up when investigating spatial QRS-T angle and all-cause death (*P* value of interaction was less than 0.01). However the interaction did not result in a significant heterogeneity in the overall analysis (*I* ^*2*^ = 28.3%, *P* = 0.175). Notably, the total number of patients in all three studies with length of follow-up less than 5 years was 1986, which was very small, leading to wide-range-covering confidence intervals. No evidence of heterogeneity was detected for stratified analyses of spatial QRS-T angle, while high level of heterogeneity was found in several stratified analyses of frontal QRS-T angle.

**Table 2:** Stratified analyses in subgroups.

Type of meta-analyses	No. of studies	Model	RR (95% CI)	*I* ^*2*^ (%)	*P_*hetero	*P*_Begg	*P_int*
**Spatial QRS-T angle predicts all-cause death (wide angle versus normal one)**							
**Type of clinical presentation**							
General population	6	Fixed	1.37 (1.29–1.46)	0	0.44	0.452	0.197
Suspected CHD	4	Fixed	1.47 (1.28–1.65)	19.3	0.293	0.734	
**No. of participants**							
>4000	5	Fixed	1.37 (1.28–1.46)	14.8	0.32	0.806	0.103
<4000	6	Fixed	1.48 (1.33–1.64)	35.2	0.173	0.707	
**Duration of follow-up**							
>5 years	8	Fixed	1.38 (1.31–1.46)	0	0.583	0.266	0.000
<5 years	3	Fixed	2.36 (1.65–2.39)	0	0.987	1	
**Spatial QRS-T angle predicts cardiac death (wide angle versus normal one)**							
**Type of clinical presentation**							
General population	5	Fixed	1.66 (1.47–1.88)	0	0.909	0.462	0.319
Suspected CHD	3	Fixed	1.81 (1.50–2.19)	6.6	0.343	0.296	
**No. of participants**							
>4000	5	Fixed	1.66 (1.47–1.88)	0	0.909	0.462	0.319
<4000	3	Fixed	1.81 (1.50–2.19)	6.6	0.343	0.296	
**Duration of follow-up**							
>5 years	7	Fixed	1.71 (1.54–1.90)	0	0.714	1	0.954
<5 years	1	–	–	–	–	–	
**Frontal QRS-T angle predicts all-cause death (wide angle versus normal one)**							
**Type of clinical presentation**							
General population	3	Random	1.29 (1.03–1.62)	83.1	0.003	1	
Suspected CHD	4	Fixed	1.74 (1.41–2.14)	45.4	0.139	1	
Heart failure	6	Random	1.51 (1.18–1.94)	64.2	0.016	1	
**No. of participants**							
>2000	4	Random	1.30 (1.11–1.51)	75.2	0.007	0.734	0.432
<2000	6	Random	1.57 (1.19–2.08)	53.2	0.06	0.454	
**Duration of follow-up**							
>5 years	3	Random	1.29 (1.03–1.62)	83	0.003	1	0.518
<5 years	7	Random	1.51 (1.21–1.88)	57.1	0.03	1	

*P_hetero*: *P* value of heterogeneity across studies; *P_Begg*: *P* value from Begg’s test; *P_int*: *P* value of interaction in subgroups. CI: confidence intervals; RR: relative risks.

When comparing a wide QRS-T angle with a normal one, notably for both spatial and frontal QRS-T angles, the RRs tended to be lower in participants from general population than those in patients with a particular disease, such as suspected CHD and heart failure (Table 2). Similarly, in studies with a larger number of participants and a longer duration of follow-up, the RRs were smaller.

Sensitivity analyses in both overall analyses and stratified analyses by omitting one study at a time showed that none of the studies substantially changed the direction of the pooled RRs. The significant heterogeneity detected in stratified analyses of frontal QRS-T angle, however, did become much smaller or even non-significant when certain individual study was omitted. For instance, in analysis of studies with number of participants less than 2000, when the study of Selvaraj *et al* was omitted, the *I* ^*2*^ and *P* value for Begg’s test changed from 53.2 and 0.06 to 27.8 and 0.24 respectively, however the direction of RR remained consistent.

### Meta-analyses of unadjusted data

We pooled data not adjusted for any confounding factors at the same time. The results were broadly similar with those from maximum-adjusted data, while there were discrepancies in amplitudes (S2 Table).

## Discussion

In our meta-analysis, a wide spatial/frontal QRS-T angle was strongly associated with a higher incidence of all-cause/cardiac death in all populations, including general population, patients with suspected CHD and patients with heart failure. Although precisely quantitative conclusions of the prognostic value of QRS-T angles might not be addressed due to the limitations in our study, a qualitative result has been defined.

To the best of our knowledge, this is the first meta-analysis conducted on QRS-T angles. QRS-T angles have been defined and studied for several decades, but intensive publications on prognostic information of QRS-T angles have not arisen until the last decade. Spatial QRS-T angle was noted earlier than frontal QRS-T angle. Spatial QRS-T angle reflects both the ventricular repolarization and depolarization vectors and thus has been considered as a potentially important ECG parameter with predictive value of the prognosis, because several variables on either repolarization or depolarization have been shown to predict cardiac morbidity and mortality [1,2,33]. Indeed in 2003, Kardys and colleagues reported that spatial QRS-T angle was a strong and independent predictor of cardiac death in an elderly population, and even stronger than any classical cardiovascular risk factors or known risk ECG variables [22]. Subsequently, a series of studies were conducted in various populations, with different number of participants and length of follow-up. Most of these studies yielded largely consistent results, although heterogeneity existed in the definitions of cut-offs and methods of categorizing. Because spatial QRS-T angle is not readily available in ECG machine currently in use, and is not familiar to most physicians, researchers begin to investigate frontal QRS-T angle, the reflection of spatial QRS-T angle on frontal plane, which is simpler to calculate. We believe that with the rapid development of automated ECG machine, the acquisition of spatial QRS-T angle would no longer puzzle physicians. It’s the good-or-bad (specificity and sensitivity) of the predictor, but not the small difference of availability, that should be considered most by clinicians once the clinical value of QRS-T angle is documented.

The population heterogeneity of these individual studies made stratified analyses in subgroup populations possible. In our study, we found that a wide QRS-T angle predicted a poor prognosis in general population, subpopulation with suspected CHD and subpopulation with heart failure. Notably, a more remarkable RR was detected in both subpopulations than in general population for both spatial and frontal QRS-T angles. Wide QRS-T angles might be associated with myocardial structure abnormity and electrophysiology alterations, and are always seen in patients with ischemia, pacing, cardiac hypertrophy and other nonischemic cardiomyopathy [17]. Indeed, subgroup populations with suspected CHD or heart failure in our study tended to have a wider QRS-T angle, while in general population, a narrower QRS-T angle was observed. Thus, a further increase of QRS-T angle on the basis of a “normal” angle which is actually wide in the subpopulations might generate a higher risk of poor prognosis than those in general population. Other stratified analyses categorized by the number of participants and the duration of follow-up were also performed. Studies with a larger number of individuals and a longer duration of follow-up generated less remarkable, but still significant RRs than their opposite categories respectively. It is not a surprise as it’s generally believed that data from studies with more participants and longer follow-ups are more credible and convincing, also in our study, more conservative.

The prognostic effect of QRS-T angles on all-cause/cardiac death might bring out their values on risk stratification, especially in patients with certain clinical presentation. In a recent meta-analysis aiming at seeking predictors of sudden cardiac death in patients with nonischemic dilated cardiomyopathy, only modest risk stratification was found in functional parameters, depolarization abnormalities, and repolarization abnormalities [33], in which QRS-T angle was not included due to lack of studies in that population [33]. Therefore, a comprehensive method of stratification combining numerous risk factors and other parameters, in which QRS-T angle might be vital, is necessary to well determine whether a patient is at higher risk. It has been commonly accepted that multivariate predictors significantly outperform individual factors in risk stratification [34]. This is because the nature of an end event is always multifactorial, but an individual predictor could only represent a single property (or pathophysiological pathway), rather than the overall pattern of performance [33,34]. Therefore, it is very difficult for an individual predictor to generate odd ratios high enough to confer meaningful prediction [34–36]. For instance, a risk score model comprising 5 clinical factors, each of which has a hazard ratio < 2, is sufficient to identify intermediate-risk patients who could gain pronounced benefits from ICD therapy [37]. QRS-T angle represents different pathophysiological pathway from conventional factors, and thus might add substantial value in the combined stratification. To support this, Strauss *et al*. screened the entire health system ECG databases in two hospitals and found that by stratification with the combination of QRS score and QRS-T angle, high-risk patients with a 1-year mortality of 8.8% to 13.9% could be identified [20]. In addition, QRS-T angle could also be used to identify relatively low-risk patients. In a study of patients with ischemic heart disease and ICD therapy, patients with a spatial QRS-T angle <100° had no event of ventricular arrhythmia after 2 years’ follow-up, and only 2% happened during further follow-up [3]. Despite all these evidences, translating QRS-T angle into clinical application needs more proofs and other studies are warranted to integrate QRS-T angle with other predictors in clinical practice.

Provided the predictive value of QRS-T angle in general population, one may ask whether it is cost-effective to perform routine screening of 12-lead ECG among subjects without a known cardiac disease. There is lack of studies evaluating the cost-effectiveness of systematic ECG screening in the field of QRS-T angle, relative evidence overwhelmingly come from trials for the prevention of sudden cardiac death in athletes [38–40]. The American Heart Association (AHA) guideline does not support universal mandatory screening with 12-lead ECG in general populations of young healthy people (12–25 years old) [39,41], because a large body of studies demonstrate that 12-lead ECG test does not provide added mortality benefit supplemental to history and physical examination [39], and the cost is far excessive for public health system [42]. Given this evidence and that QRS-T angle was less predictive in general population in our study (RR < 2); we propose that mandatory screening with 12-lead ECG in the general population is unlikely to be cost-effective. Instead, it is more reasonable to take the advantage of the prognostic value of QRS-T angles in targeted populations (particularly populations with cardiovascular diseases) [39], in which 12-lead ECG is itself mandatory and the added benefits of QRS-T angles in risk stratification could be realized.

### Study limitations

Several limitations should be acknowledged in our study. First, studies in our meta-analysis were conducted in different populations. Although this made stratified analyses in subgroup populations possible, the number of studies in each subpopulation was relatively limited. Besides, the definitions of subgroup populations were not uniform across studies. For instance, in patients with suspected CHD, some patients were enrolled with a symptom of acute ischemic chest pain; others were those undergoing a clinically indicated bicycle stress-test, or those with clinically diagnosed CHD. Thus, a phrase of “suspected CHD” was used in our study to indicate the potential heterogeneity.

Second, heterogeneity in the definitions of the cut-offs of QRS-T angles existed. As the population in each study varies, and QRS-T angles change with the pathophysiological status of the participants, the normal range of this angle in each population is different. Meanwhile, two methods of categorizing were used among these studies. Combining results from these two methods might bring in bias to our study. We conducted separate analyses to minimize this kind of bias, and both analyses yielded similar results. All these limitations made precise quantitative evaluation of prognostic value of QRS-T angle impossible. However, qualitative conclusions can still be addressed, evidenced by results from separate analyses. The consistency between adjusted and unadjusted analyses also reinforces the validity of our present findings.

Third, the extents of adjustment for confounding factors were not uniform across studies. The confounding factors for which were adjusted included demographic and risk factors (age, gender, presence of hypertension, diabetes mellitus, *etc*.), ECG parameters (STT abnormity, QRS duration, QTc interval, *etc*.), and drug use (diuretics, blockers, calcium channel blockers, *etc*.). Adjustments for certain confounders were absent in some studies, which could bring biases into individual studies. For instance, lack of adjustment for antihypertensive drugs might overlook the status of blood pressure controlling, which could subsequently affect the baseline QRS-T angles recorded in individual studies [43]. To maximally limit this kind of bias, we extracted the maximum-adjusted data from each study, and thus the bias is unlikely to be large.

Fourth, most of the individual studies were carried out in Europe, thus the results of this analysis might not be generalizable to other ethnic populations. Further studies in these populations are needed for the final determination.

## Conclusions

The present meta-analysis of all the evidence available demonstrates that both spatial and frontal QRS-T angles carry promising prognostic information on all-cause mortality in general population, in patients with suspected CHD and patients with heart failure. A wide spatial QRS-T angle also predicts a higher incidence of cardiac death. Given the great predictive value of QRS-T angles on all-cause/cardiac mortality, a combing stratification strategy in which QRS-T angle is of vital importance might be expected in near future. Of course, more solid evidence from well-designed studies is necessary.

## Supporting Information

S1 Checklist PRISMA checklist. (DOC)

S1 Table Study quality, endpoints and confounders included in adjusted estimates of studies. (DOC)

S2 Table Unadjusted results of meta-analyses for spatial/frontal QRS-T angle and all-cause mortality/cardiac mortality. (DOC)
