# Supplementary material for: Spatial/Frontal QRS-T Angle Predicts All-Cause Mortality and Cardiac Mortality: A Meta-Analysis
Source: PLoS One. 2015 Aug 18;10(8):e0136174. doi: 10.1371/journal.pone.0136174 (PMC4540436; doi:10.1371/journal.pone.0136174)
Supplement: S1 Table — (DOC) [file pone.0136174.s002.doc]

**S1 Table.** Study quality, endpoints and confounders included in adjusted estimates of studies.

| **Author** | **Year** | **Study quality (Newcastle-Ottawa Scale)** | | | | **Endpoints** | **Confounders included in adjusted estimates** |
| --- | --- | --- | --- | --- | --- | --- | --- |
|  |  | Selection | Comparability | Outcome/ exposure | Total score |  |  |
| Kardys | 2003 | ******** | ****** | ******* | 9 | Cardiac death, nonfatal cardiac events, sudden death and all-cause mortality | Age, sex, current smoking, body mass index >25 kg/m2, hypertension, total cholesterol/HDL ratio >7.2, diabetes mellitus, history of angina pectoris and history of myocardial infarction, and other ECG parameters |
| de Torbal | 2004 | ******** | ****** | ******* | 9 | All-cause mortality | Age, gender, history of hypertension, diabetes mellitus, myocardial infarction, heart failure and coronary revascularization, and electrocardiogram variables. |
| Yamazaki | 2005 | ******** | ***** | ******* | 8 | Cardiovascular death | age, gender, and heart rate |
| CHS | 2006 | ******** | ****** | ******* | 9 | CHD mortality and all-cause mortality | Age, gender, race, body mass index, hypertensive status, and diabetes mellitus and drug use (diuretics, blockers, antiarrhythmic classes 1a to 1c and 3, and calcium channel blockers) |
| WHI-1 | 2006 | ******** | ****** | ******* | 9 | Incident CHD and CHD mortality | Age, ethnicity, systolic blood pressure, and body mass index, smoking, hormone therapy use at baseline, self-report of the use of cholesterol-lowering drugs, self-report of diabetes control, the use of cardioactive drugs (antiarrhythmic drugs, calcium channel blockers, β-blockers, diuretics, antidepressants, or psychotherapeutic drugs), and mulitiple ECG variables. |
| WHI-2 | 2006 | ******** | ****** | ******* | 9 | Incident CHF and all-cause mortality | Age, ethnicity, systolic blood pressure, and body mass index, smoking, hormone therapy use at baseline, self-report of the use of cholesterol-lowering drugs, self-report of diabetes control, the use of cardioactive drugs (antiarrhythmic drugs, calcium channel blockers, β-blockers, diuretics, antidepressants, or psychotherapeutic drugs), and mulitiple ECG variables. |
| ARIC | 2007 | ******** | ****** | ******* | 9 | Incident CHD ( includes incident MI, electrocardiographic MI, nonfatal CHD, or fatal CHD) and all-cause mortality | Gender, race, body mass index, education, family history of stroke, family history of CHD, smoking status, alcohol use, asthma, cancer, diabetes mellitus, hypertension, Rose angina, Rose intermittent claudication, sport index, forced expiratory volume in 1 second, high-density lipoprotein, total triglycerides, total cholesterol, systolic blood pressure, diastolic blood pressure, hematocrit, white blood cells, total calories, dietary cholesterol, ankle– brachial index, baseline fasting blood glucose, insulin, creatinine, fibrinogen, uric acid, STT abnormity, QRS/T angles and T-wave axis |
| DEFINITE | 2008 | ******** | ****** | ****** | 8 | A composite of total mortality, appropriate implantable cardioverter-defibrillator shock, or resuscitated cardiac arrest; all-cause mortality | Treatment group, age, gender, QRS duration, left bundle-branch block, left ventricular ejection fraction, New York Heart Association class III, atrial fibrillation, and diabetes mellitus |
| Borleffs | 2009 | ******** | ****** | ****** | 8 | All-cause mortality and ventricular arrhythmia | Age, sex, LVEF, and QRS duration. |
| Lipton | 2009 | ******** | ****** | ******* | 9 | All-cause mortality and sudden cardiac mortality | Age, sex, history of diabetes, history of MI, history of heart failure, smoking, hypertension, hypercholesterolemia, peak wall motion score, and DSE result for ischemia. |
| Rubulis | 2010 | ******** | ****** | ****** | 8 | Cardiovascular death | Age, sex, left ventricular (LV) hypertrophy, and QRS duration |
| FINCAVAS | 2011 | ******** | ****** | ****** | 8 | All-cause mortality, cardiac mortality, and sudden cardiac death. | Sex, HRR, maximum heart rate and baseline ST segment depression, usage of beta-blockers |
| Aro | 2012 | ******** | ****** | ******* | 9 | Death from arrhythmia, all-cause mortality and non-arrhythmic cardiac mortality | Age, sex, smoking, heart rate, systolic blood pressure, chronotropic medication, QRS duration, electrocardiogram signs of coronary artery disease |
| Lown | 2012 | ******** | ****** | ****** | 8 | All-cause mortality | Age, heart failure, previous acute myocardial infarction, heart rate, systolic blood pressure, ST-segment depression on admission electrocardiography, creatinine, elevated cardiac markers, and inpatient percutaneous coronary intervention |
| NHANES III | 2012 | ******** | ****** | ******* | 9 | All-cause mortality and cardiac death | Age, race/ethnicity, sex, body mass index, physical inactivity, current smoking, systolic blood pressure, antihypertensive medication use, heart rate, diabetes, total and high-density lipoprotein cholesterol, cholesterol lowering medication use, C-reactive protein ≥ 3 mg/L, reduced estimated glomerular filtration rate, albuminuria, and a history of stroke. |
| de Bie | 2013 | ******** | ****** | ****** | 8 | All-cause mortality and sudden cardiac mortality | Age, type of dialysis, coronary artery disease, diabetes mellitus, not in sinus rhythm, heart rate, QRS duration, and presence of Q-waves |
| Gotsman | 2013 | ******** | ****** | ****** | 8 | All-cause mortality, cardiac-related hospitalization | Age, gender, ischemic heart disease, hypertension, atrial fibrillation, body mass index, pulse, serum hemoglobin, sodium, estimated glomerular filtration rate, and urea levels |
| MADIT II | 2013 | ******** | NA | ****** | NA | All-cause mortality and VT/VF | Not available (conference abstract) |
| Strauss | 2013 | ******** | ― | ****** | 6 | All-cause mortality | No adjustment |
| Laukkanen | 2014 | ******** | ****** | ******* | 9 | Sudden death and all-cause mortality | Age, alcohol consumption, cigarette smoking, serum low- and high-density lipoprotein cholesterol, systolic blood pressure, type 2 diabetes, BMI, high-sensitivity C-reactive protein, previous myocardial infarction, and cardiorespiratory fitness. |
| Raposeiras-Roubín | 2014 | ******** | ****** | ****** | 8 | All-cause mortality and cardiac death | Age, diabetes mellitus, previous coronary artery disease, previous heart failure, peripheral artery disease, STEMI, LVEF, drug use and ECG variables |
| Selvaraj | 2014 | ******** | ****** | ****** | 8 | All-cause mortality, cardiovascular hospitalization | Age, sex, body mass index, diabetes, atrial fibrillation, coronary artery disease, estimated glomerular filtration rate, amiodarone use, diuretic use, QRS duration, QTc interval, and BNP |

*Abbreviations:* ARIC: the Atherosclerosis in Communities Study; BNP: brain natriuretic peptide; CHD: coronary heart disease; CHF: congestive heart failure; CHS: the Cardiovascular Health Study; DEFINITE: the Defibrillators in Nonischemic Cardiomyopathy Treatment Evaluation; DSE: dobutamine stress echocardiography; FINCAVAS: The Finnish Cardiovascular Study; LVEF: left ventricular ejection fraction; MADIT II: the Multicenter Automatic Defibrillator Implantation Trial II; NHANES III: the Third National Health and Nutrition Examination Survey; SMART: the Strategies for the Management of Antiretroviral Therapy study STEMI: ST-segment elevation myocardial infarction; WHI: The Women’s Health Initiative.
