# Supplementary material for: Spatial/Frontal QRS-T Angle Predicts All-Cause Mortality and Cardiac Mortality: A Meta-Analysis
Source: PLoS One. 2015 Aug 18;10(8):e0136174. doi: 10.1371/journal.pone.0136174 (PMC4540436; doi:10.1371/journal.pone.0136174)
Supplement: S2 Table — (DOC) [file pone.0136174.s003.doc]

**S2 Table.** Unadjusted results of meta-analyses for spatial/frontal QRS-T angle and all-cause mortality/cardiac mortality.

|  |  | Frontal QRS-T angle | Spatial QRS-T angle | Spatial QRS-T angle |
| --- | --- | --- | --- | --- |
|  |  | All-cause death | All-cause death | Cardiac death |
| Dual comparison | RR (95% CI) | 2.00 (1.39, 2.88) | 2.31 (2.07, 2.59) | 3.18 (1.66, 6.09) |
|  | No.of studies | 4 | 4 | 2 |
|  | No.of individuals | 12047 | 20474 | 1484 |
|  | *I^2^*_heterogeneity | 86.3% | 5.6% | 49.2% |
|  | *P*_heterogeneity | 0.000 | 0.365 | 0.161 |
|  | *P*_Begg | 0.734 | 0.734 | 1.000 |
| Abnormal vs. normal | RR (95% CI) | 2.26 (1.68, 3.04) | 2.49 (1.67, 3.71) | 3.61 (1.90, 6.86) |
|  | No.of studies | 2 | 4 | 3 |
|  | No.of individuals | 2219 | 17794 | 15533 |
|  | *I^2^*_heterogeneity | 0.0% | 97.3% | 96.4% |
|  | *P*_heterogeneity | 0.826 | 0.000 | 0.000 |
|  | *P*_Begg | 1.000 | 0.734 | 0.296 |
| Borderline vs. normal | RR (95% CI) | 1.55 (1.18, 2.04) | 1.67 (1.18, 2.38) | 1.90 (1.13, 3.17) |
|  | No.of studies | 2 | 4 | 3 |
|  | No.of individuals | 2219 | 17794 | 15533 |
|  | *I^2^*_heterogeneity | 0.0% | 96.3% | 92.5% |
|  | *P*_heterogeneity | 0.458 | 0.000 | 0.000 |
|  | *P*_Begg | 1.000 | 1.000 | 0.296 |
| Combined analyses | RR (95% CI) | 1.90 (1.49, 2.42) | 2.29 (1.66, 3.15) | 2.80 (1.51, 5.20) |
|  | No.of studies | 6 | 8 | 5 |
|  | No.of individuals | 14266 | 38268 | 17017 |
|  | *I^2^*_heterogeneity | 80.5% | 96.3% | 95.0% |
|  | *P*_heterogeneity | 0.000 | 0.000 | 0.000 |
|  | *P*_Begg | 0.707 | 0.902 | 0.806 |

Meta-analyses were from separated comparisons by two methods of categorizing. In the first method, Spatial QRS-T angle was divided into two groups and comparison between these two groups was named “dual comparison”. In the second method, spatial QRS-T angle was categorized into three groups: normal, borderline and abnormal, results were from comparison between abnormal and normal, and from comparison between borderline and normal.

*P_heterogeneity*: *P* value of heterogeneity across studies; *P_Begg*: *P* value from Begg’s test; *P_int*: *P* value of interaction in subgroups. CI: confidence intervals; RR: relative risks.
